# Targeting the non-canonical roles of PCNA modifies and increases the response to targeted anti-cancer therapy

**DOI:** 10.18632/oncotarget.27267

**Published:** 2019-12-31

**Authors:** Caroline K. Søgaard, Anala Nepal, Voin Petrovic, Animesh Sharma, Nina-Beate Liabakk, Tonje S. Steigedal, Marit Otterlei

**Affiliations:** ^1^ Department of Clinical and Molecular Medicine, Norwegian University of Science and Technology (NTNU), Trondheim, Norway; ^2^ Clinic of Surgery, St. Olavs Hospital, Trondheim University Hospital, Trondheim, Norway; ^3^ Proteomics and Modomics Experimental Core Facility (PROMEC), NTNU, Trondheim, Norway; ^4^ APIM Therapeutics A/S, Trondheim, Norway

**Keywords:** APIM, multi-targeting therapy, signaling pathways, autophagy, cancer

## Abstract

Receptor tyrosine kinases (RTKs), such as HER2 and/or EGFR are important therapeutic targets in multiple cancer cells. Low and/or short response to targeted therapies are often due to activation of compensatory signaling pathways, and therefore a combination of kinase inhibitors with other anti-cancer therapies have been proposed as promising strategies. PCNA is recently shown to have non-canonical cytosolic roles, and targeting PCNA with a cell-penetrating peptide containing the PCNA-interacting motif APIM is shown to mediate changes in central signaling pathways such as PI3K/Akt and MAPK, acting downstream of multiple RTKs. In this study, we show how targeting PCNA increased the anti-cancer activity of EGFR/HER2/VEGFR inhibition *in vitro* as well as *in vivo.* The combination treatment resulted in reduced tumor load and increased the survival compared to either single agent treatments. The combination treatment affected multiple cellular signaling responses not seen by EGFR/HER2/VEGFR inhibition alone, and changes were seen in pathways determining protein degradation, ER-stress, apoptosis and autophagy. Our results suggest that targeting the non-canonical roles of PCNA in cellular signaling have the potential to improve targeted therapies.

## INTRODUCTION

Receptor tyrosine kinases (RTKs), such as epidermal growth factor receptor (EGFR), human epidermal growth factor receptor 2 (HER2) and endothelial growth factor receptor (VEGFR), and the downstream mitogen-activated protein kinase (MAPK) and phosphoinositide 3-kinase (PI3K)/Akt pathways are often de-regulated in multiple solid tumors, e.g. breast, lung, bladder and colon cancer. These proteins/pathways support proliferation, survival and development of drug resistance, and inhibitors against these kinases have been, or are under current investigations [1–3]. However, drug resistance is the major obstacle for targeted therapies in general as cells have robust mechanisms to circumvent the effects of specific inhibitors. Combination treatments attempt to overcome this problem by targeting several pathways simultaneously [4]. Thus, although VEGFR and EGFR inhibitors have failed to improve overall survival, they are still considered promising for combination therapies [5–8].

Proliferating cell nuclear antigen (PCNA) is best known for its canonical scaffolding roles in DNA replication, DNA repair and DNA damage tolerance [9, 10]. However, non-canonical roles in regulation of apoptosis, immune evasion, glycolysis and cellular signaling have recently been discovered [11–17]. PCNA may interact with more than 500 proteins through either of the two PCNA-interacting motifs; the PCNA-interacting peptide (PIP)-box or AlkB homologue 2 PCNA-interacting motif (APIM) [18, 19]. Approximately 90 of the potential PCNA-interacting proteins are signaling protein kinases (http://tare.medisin.ntnu.no/pcna/index.php), and several APIM-containing proteins are involved in the MAPK and PI3K/Akt pathways acting downstream of VEGFR and EGFR [20]. A cell-penetrating APIM-containing peptide (ATX-101) can disrupt PCNA from interacting with APIM-containing proteins [17, 21, 22]. ATX-101 may therefore simultaneously impair several signaling pathways considered therapeutic targets in multiple cancers. ATX-101 has previously been shown to enhance the efficacy of different chemotherapeutics in *in vivo* cancer models [23-25], to inhibit mutagenesis by impairing DNA translesion synthesis (TLS) [22] and to modulate the PI3K/Akt and MAPK pathways [17]. Thus, ATX-101 could potentially both enhance, and prolong the efficacy of targeted therapies.

In this study, we examined the effects of combining ATX-101 with an EGFR/HER2/VEGFR inhibitor (AEE788) *in vitro* and *in vivo* in an orthotopic syngeneic HER2-/progesterone receptor - (PR-), estrogen receptor + (ER+)/EGFR+ mixed luminal/basal breast cancer mouse model [26-28]. We detected a significant reduced tumor volume in combination treated mice compared to single agent treated mice. Alterations in signaling proteins detected 24 hours after treatments, suggested increased apoptosis, ER stress and autophagy, in addition to reprogrammed signaling downstream of EGFR/HER2/VEGFR in the combination group. Our results are supportive of cytosolic roles of PCNA, and suggest that targeting PCNA could be a novel strategy to increase anti-cancer efficacy of targeted therapies.

## RESULTS

### ATX-101 increases the anti-cancer efficacy of an EGFR/HER2/VEGFR inhibitor

**Figure 1:**
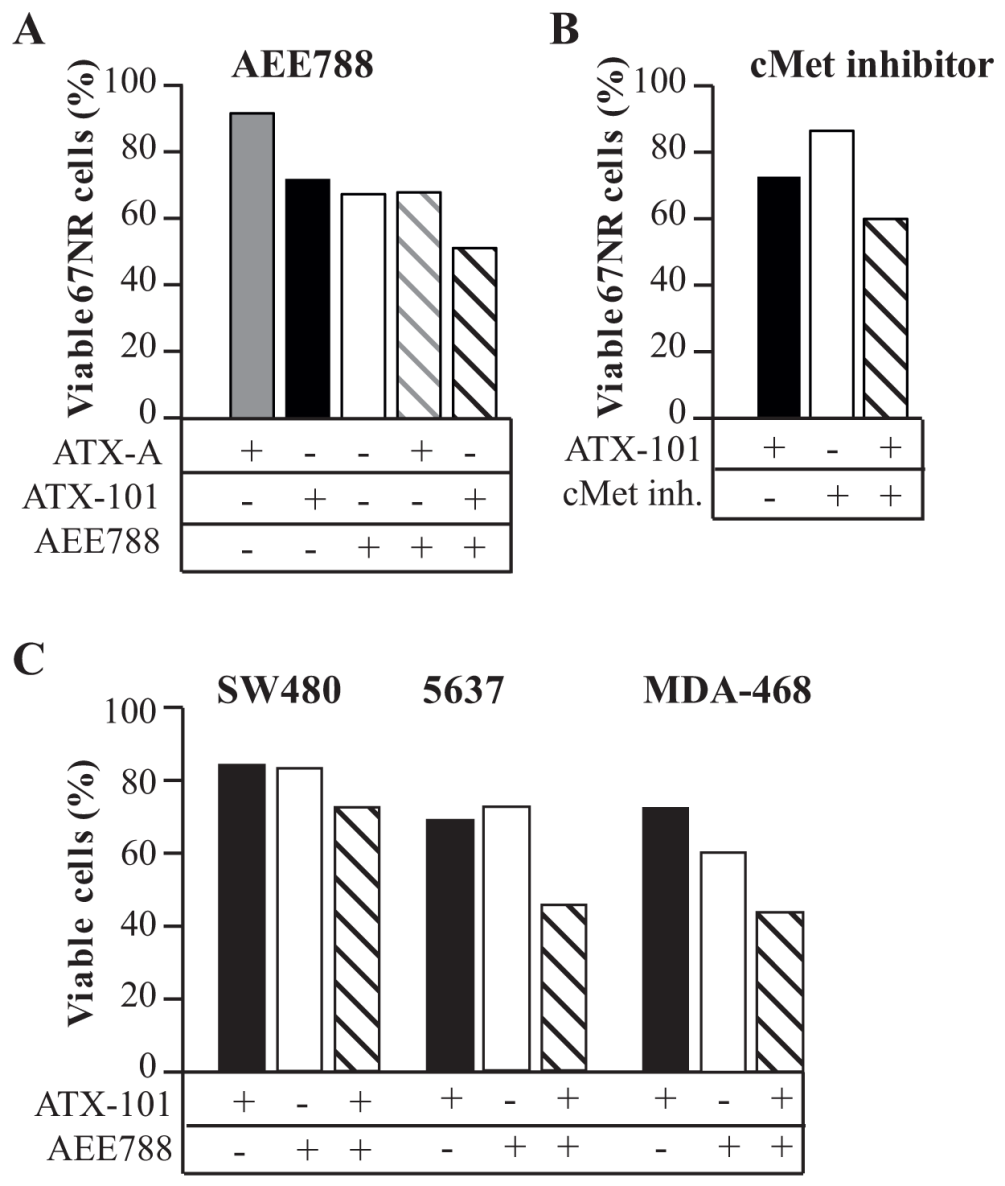
ATX-101 enhances the efficacy of RTK inhibition Cell viability after continuous exposure to the indicated treatment for 3 days relative to untreated control. One representative experiment out of three biological replicas with the same trends are shown. **(A)** 67NR mouse breast cancer cells treated with ATX-101 (6 μM), ATX-A (6 μM, mutated APIM-peptide), AEE788 (1 μM) or the combination of these. **(B)** 67NR mouse breast cancer cells treated with ATX-101 (6 μM), cMet inhibitor (PHA-665752) (2 μM) or the combination of these. **(C)** SW480 colon cancer cells treated with ATX-101 (8 μM), AEE788 (1 μM) or the combination of these. 5637 human bladder cancer cells treated with ATX-101 (12 μM), AEE788 (0.5 μM) or the combination of these. MDA-468 human breast cancer cells treated with ATX-101 (4 μM), AEE788 (1 μM) or the combination of these.

Resistance to targeted therapy limits the therapeutic efficacy. Because PCNA has been linked to regulation of the PI3K/Akt pathway [17, 24], we therefore examined if the PCNA targeting peptide ATX-101 could increase the efficacy of AEE788, an inhibitor of EGFR/HER2/VEGFR. The ATX-101/AEE788 combination significantly reduced the percentage of viable 67NR cells compared to single treatments *in vitro* (Figure 1A). We have previously shown that the biological effects of ATX-101 depends on its PCNA affinity, and that a peptide with reduced PCNA binding affinity (ATX-A) has no biological effect [17, 18, 21, 22]. Also in this study, ATX-A had much lower effect on viability than ATX-101, and importantly did not enhance the growth inhibiting effect of AEE788 (Figure 1A). This supports that the biological effect detected is mediated by ATX-101 interacting with PCNA, blocking PCNA-protein interactions. The effect of ATX-101 is likely mainly mediated via PCNA´s role in regulation of the PI3K/Akt pathways downstream of receptor tyrosine kinases (RTKs), because ATX-101 also reduced the viability of 67NR cells when combined with an inhibitor of cMet (Figure 1B). cMET is an RTK often overexpressed as a response to drugs targeting EGFR, thereby contributing to acquired resistance. The activity of ATX-101 is not specific for the 67NR cells as ATX-101 also enhanced the effect of AEE788 in three other human cancer cell lines overexpressing EGFR; the human colon cancer cell line SW480, the human bladder cancer cell line 5637 and the human breast cancer cell line MDA-468 (Figure 1C).

**Figure 2:**
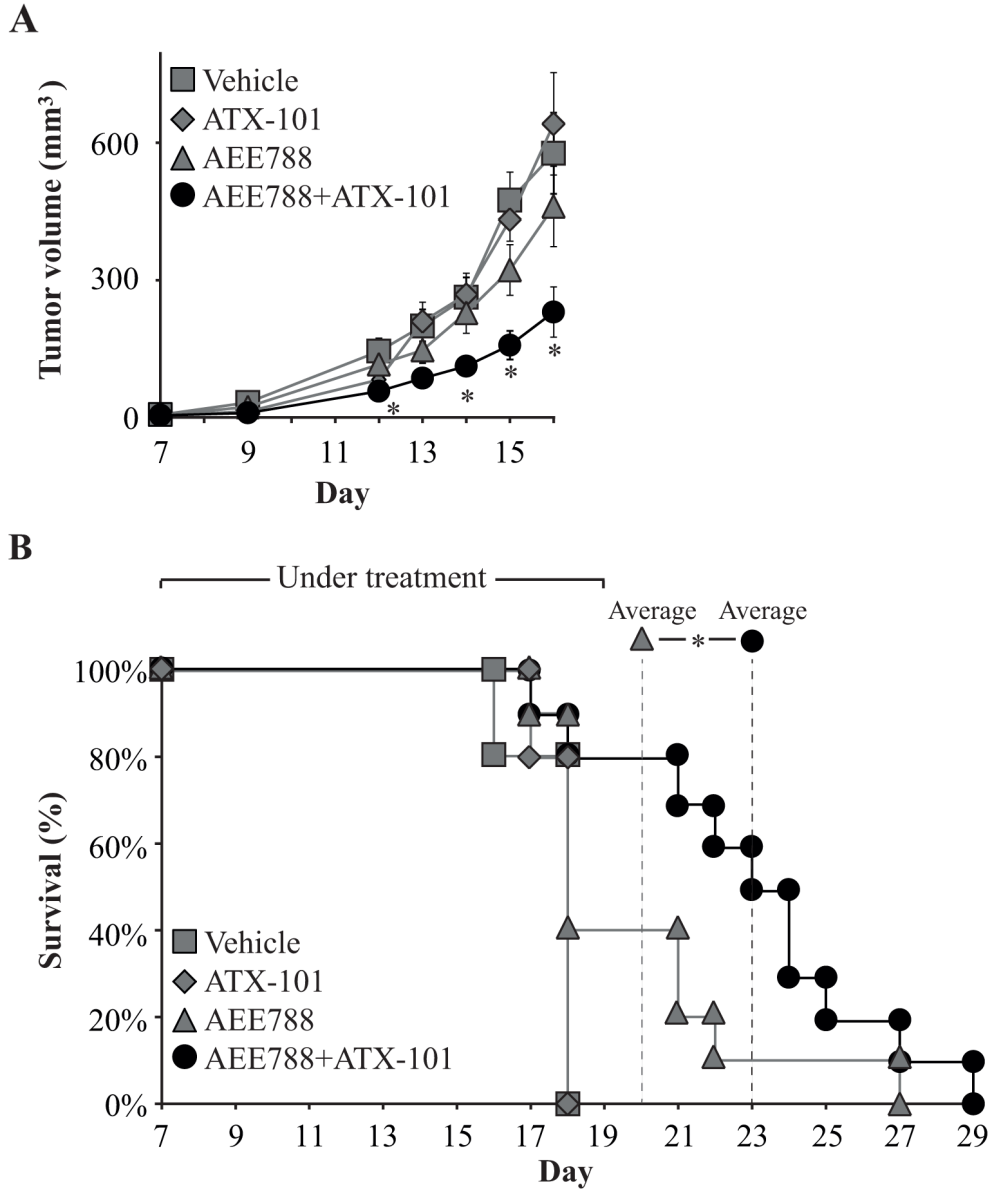
Combining AEE788 with ATX-101 improves treatment efficacy **(A)** Viability of 67NR mouse breast cancer cells after continuous exposure to ATX-101 (6 μM), AEE788 (1 μM) or the combination of these at 72h. Percentage viabilities are relative to untreated control. The average viabilities ±SD (n=4 biological replica) are displayed. Significant differences (^*^p<0.05) between single agent and combination treated groups were calculated by student t-test (two-sided, paired). **(A)** Tumor volume of 67NR tumor-bearing mice (67NR cells implanted at day 0) treated 3x/week from day 7 with vehicle (97% PEG300 in DMSO, n=5), ATX-101 (6 mg/kg net peptide, n=5), AEE788 (25 mg/kg, n=10) and ATX-101/AEE788 in combination (n=10). The average tumor volumes ± SEM are displayed. Significance between AEE788 single agent and ATX-101/AEE788 combination treated groups were calculated by student t-test (two-sided, unpaired)(^*^, p<0.05, day 12 and day 14-16). **(B)** Overall percentage survival of 67NR tumor-bearing mice shown in (A). Treatments were stopped at day 19, at the AEE788 and combination treated groups kept to study overall survival. The average number of days of survival for the AEE788 and combination treated groups are marked by dashed lines, and the significance between these two groups calculated by student t-test (two-sided, unpaired) (^*^, p=0.05).

Next, we used an orthotopic, immunocompetent mouse breast cancer model to study the effect of the combination therapy *in vivo.* This model has previously been used for examining the anticancer effects of AEE788 [7]. We found that only the mice treated with the ATX-101/AEE788 combination had a significant reduced tumor volume compared to vehicle treated mice. Importantly, the combination treated group had significantly lower tumor volume at day 12, 14-16 compared to the AEE788 single treated group (Figure 2A, asterix). The vehicle and the ATX-101 single agent treated groups reached their maximum accepted tumor burden and were terminated at day 16-18 after inoculation. We therefore stopped treating the AEE788 and ATX-101/AEE788 combination groups at day 19, but kept these two groups to study overall survival. Overall survival significantly increased for the combination treated group compared to the AEE788 treated group, with an average number of days increasing from19.8 for the AEE788 single agent treated group to 23.0 for the combination treated group (Figure 2B).

### ATX-101/AEE788 combination re-programs the kinome compared to AEE788 alone

**Figure 3:**
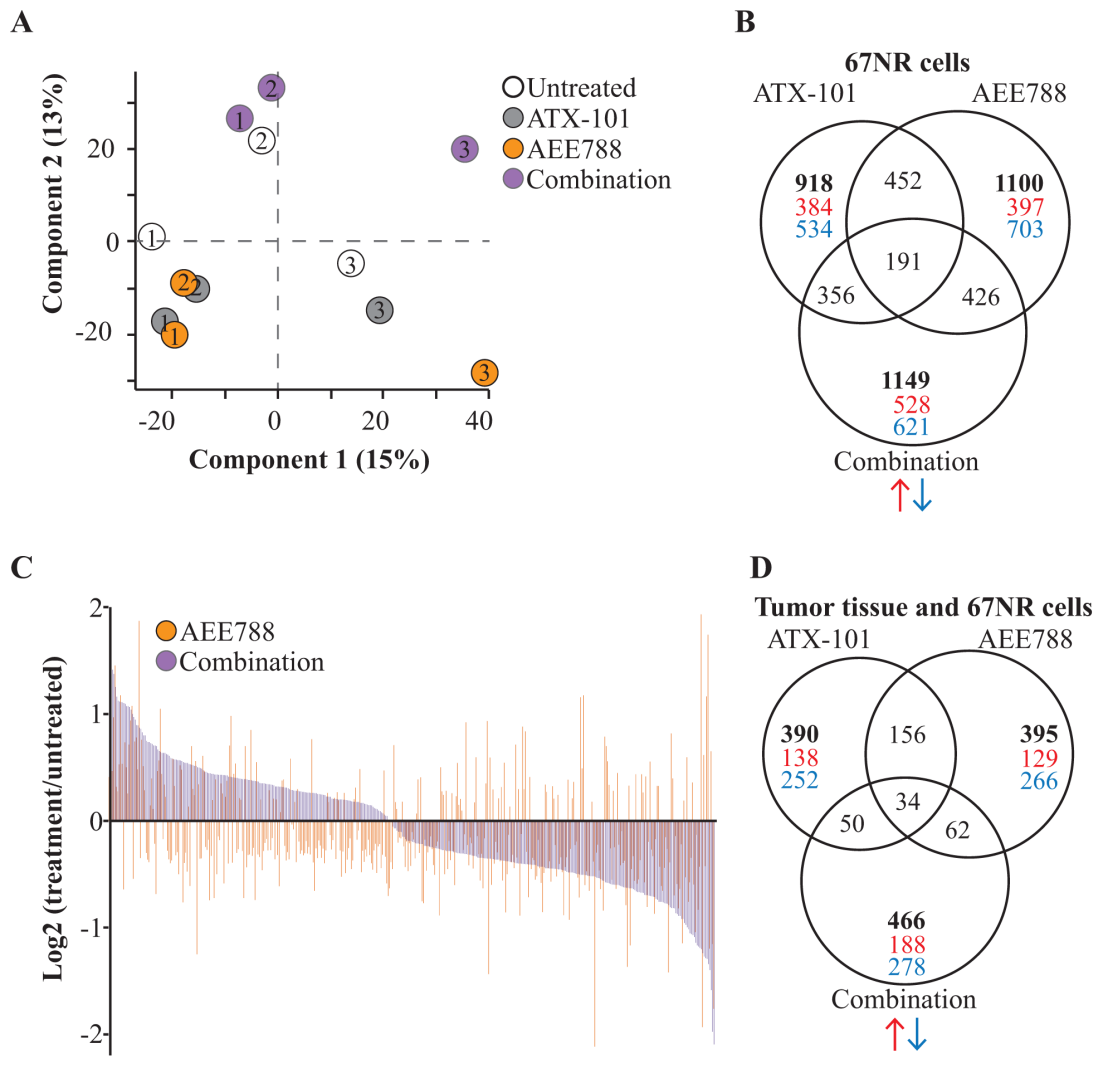
AEE788/ATX-101 combination alters cellular signaling compared to single agent treatments MIB-assay (a kinome enrichment assay) analysis of 67NR cells untreated or treated with ATX-101 (6 μM), AEE788 (1 μM) or the combination of these for 24h (n=3 for each condition). **(A)** Principal component analysis (PCA) plot displaying general differences between the samples. Number indicates the different biological replicates. Component 1 explains variance in biological replicates and component 2 explains variance in treatment groups. **(B)** Venn diagram of number of significantly changed proteins in each treatment group relative to untreated control (Wilcoxon signed-rank test, p<0.25, total number of changed proteins (bold), downregulated (blue), upregulated (red)). **(C)** Log2 transformed values of significant changed proteins relative to untreated control detected in both the AEE788 group and combination group. **(D)** Venn diagram of significantly changed proteins in 67NR cells treated *in vitro* showing the same trend in tumor tissue isolated from 67NR tumor-bearing mice. Tumor tissue were harvested 24h after the third treatment with vehicle (97% PEG300 in DMSO, n=6), ATX-101 (6 mg/kg net peptide, n=6), AEE788 (25 mg/kg, n=7) and ATX-101/AEE788 (n=7) in combination.

To unravel the underlying mechanisms of the combination treatment, we examined its effects on cellular signaling in *in vitro* treated cells and in harvested tumor tissue. We harvested 67NR cells treated *in vitro* 24 hours after treatment, and used a variant of a MS-based MIB-assay to pull down signaling proteins via binding to immobilized kinase inhibitors [29]. A PCA-plot of the proteins enriched in the different groups showed that the largest variations in the data sets could be explained by variation in biological replica (component 1, 15% variance) and treatment conditions (component 2, 13% variance). ATX-101 and AEE788 single treated groups clustered separately from the combination treated group (Figure 3A).

We detected significant changes in more than 900 proteins after ATX-101 treatment and 1100 proteins after AEE788 and combination treatments. Approximately half of the significantly changed proteins in combination group were unique for this group and not significantly changed in either of the single agent groups (Figure 3B, data deposited to PRIDE, PXD011044). The majority of proteins detected in both AEE788 and combination treated groups either showed opposite changes, i.e. increased in AEE788 and reduced in combination or vice versa, or a larger increase or reduction in the combination compared to the AEE788 group (Figure 3C), thus supporting a systemic effect after addition of ATX-101 to the AEE788 treatment.

When performing the MIB-assay on tumor tissue harvested from 6 mice/group 24 hours after the third treatment (day 13/14), we found that the variations within each treatment group were larger in the protein extracts from the tumor tissues than from the biological replicas of the *in vitro* treated cancer cell line. This is as expected as large heterogeneousness in the tissues including tumor sizes, and thus drug distribution, and content of non-tumor cells are seen. We were therefore not able to detect any significant changes between the different *in vivo* treatment groups. However, in the tumor tissues we observed the same trends as in 35-40% of the significantly altered proteins detected in the *in vitro* treated cells (Figure 3D).

### Combination therapy mainly affects EGFR/HER2/VEGFR signaling and protein degradation

**Figure 4:**
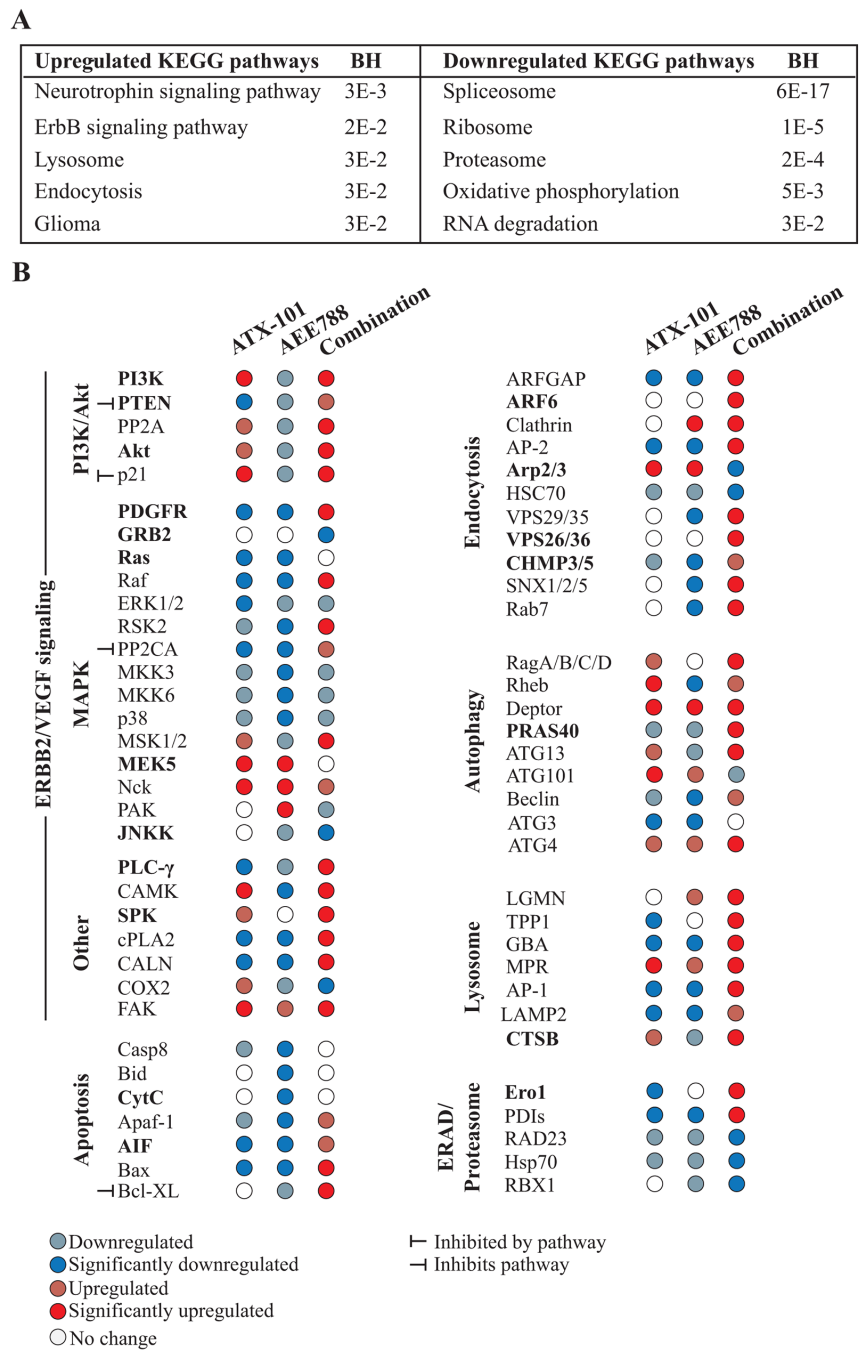
Multiple signaling proteins are differentially affected by the combination treatment than single AEE788 or ATX-101 treatments Analysis of proteins with significant changed levels in 67NR cells treated for 24h with ATX-101 (6 μM) and AEE788 (1 μM) alone or in combination, relative to untreated control, as identified by the MIB-assay. **(A)** Functional enrichment analysis of proteins identified in combination treated group. Top five significant up –and downregulated KEGG pathways (ranked by Benjamini-Hochberg (BH)) identified using the database for annotation, visualization and integrated discovery (DAVID) are displayed. **(B)** Upregulated (red) and downregulated (blue) proteins after ATX-101 and AEE788 single and combination treatments. Proteins involved in pathways often impaired in breast cancer are displayed. Proteins in bold had the same expression pattern in tumor tissue harvested from ATX-101, AEE788 and combination treated 67NR tumor-bearing mice.

Functional annotation analysis (KEGG) of the significantly altered proteins in the combination group pointed towards increased HER2 (=ErbB2) signaling, increased endocytosis and changes in protein degradation (lysosome (up), proteasome (down), RNA degradation (down)) (Figure 4A).

Analysis of the signaling pathways downstream of EGFR/HER2/VEGFR the main targets of AEE788, indicated as expected that most proteins participating in these pathways were reduced in cells treated with AEE788 and with ATX-101 as single agents, while somewhat unexpectedly they were increased in the combination treated group (Figure 4B). These pathways are generally associated with increased cellular proliferation and survival; however, we simultaneously detected an increased pull down of pro-apoptotic proteins not seen in the single agent treated groups. For example, upon treatment with AEE788 only, the key pro-apoptotic regulators Bax, Bid, Apaf-1 and CytC were downregulated, while the combination treatment led to an increase in Bax and Apaf-1, and abolished the reduction seen in CytC and Bid (Figure 4B). These alterations support increased apoptosis with the ATX-101/AEE788 combination and fits well with the reduction in tumor volume observed *in vivo*.

The main reasons for increased apoptosis are elusive and likely involve multiple pathways; however, our data suggest that the combination treatment induces ER-stress and at the same time reduces ER-associated degradation (ERAD), which collectively could explain increased apoptosis. ERO1 and PDIs, both which targets proteins for degradation, were upregulated only in the combination treated cells, while RAD23, Hsp70 and RBX1 (ERAD proteins) were more downregulated in the combination group compared to the single treatment groups (Figure 4B).

Cumulatively, these data suggest that combination with ATX-101 re-programs the AEE788 effects on these breast cancer cells and increases the anti-cancer efficacy of AEE788.

### ATX-101/AEE788 therapy increases the autophagic flux

**Figure 5:**
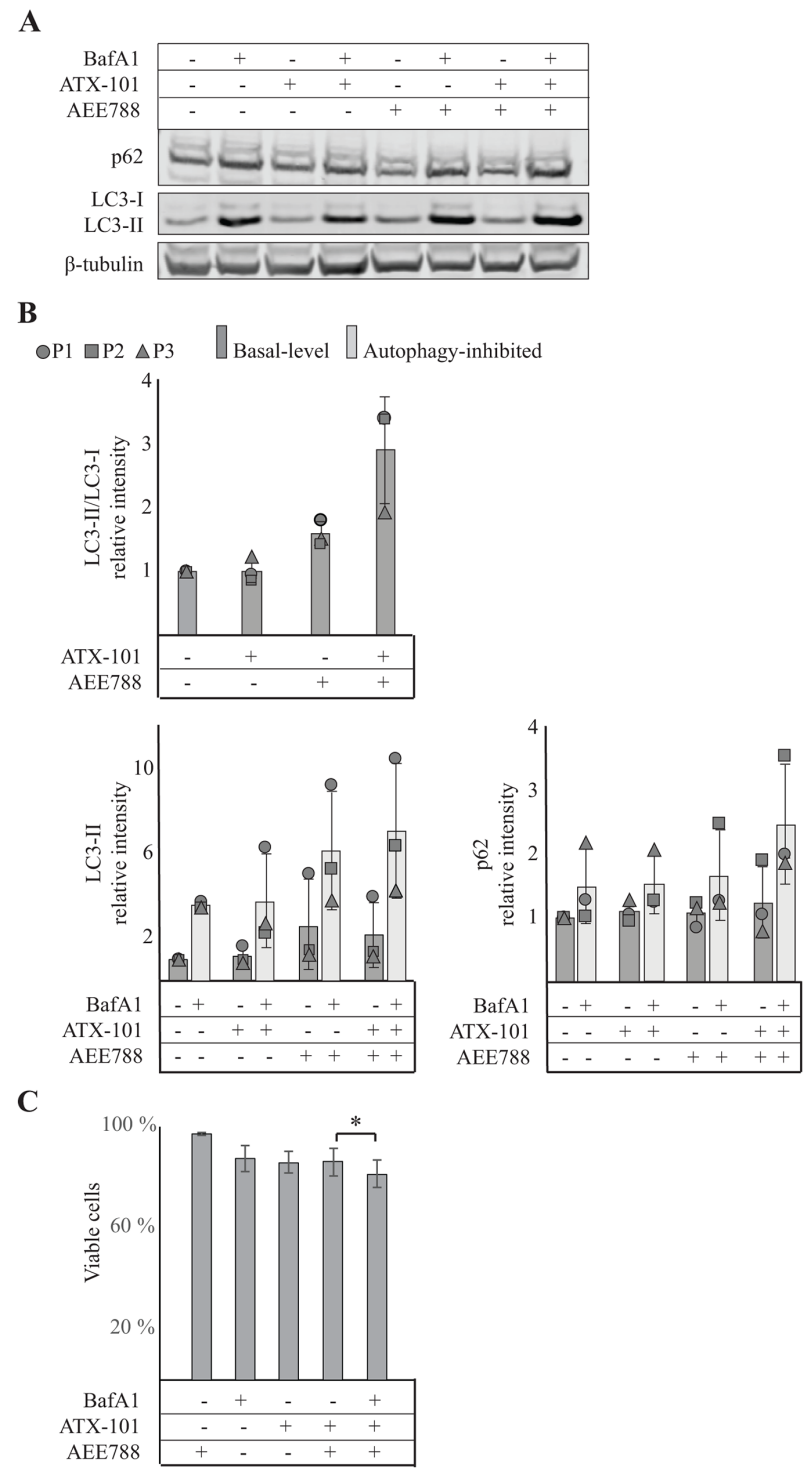
Combining ATX-101 with AEE788 treatment enhances autophagy Analysis of autophagy in 67NR cells treated for 24h with vehicle, ATX-101 (6 μM) AEE788 (1 μM), and ATX-101/AEE788 combination. **(A)** Detection of p62, LC3 and β-tubulin by western blot analysis. Each treatment condition was also exposed to the autophagy inhibitor bafilomycin 1A (10 nM, 12h). Data displayed are from one representative experiment out of three biological replicates. **(B)** Protein intensities normalized against β-tubulin and relative to untreated control. Data are presented as average ± SD (n=3). **(C)** Viability of 67NR cells after continuous exposure to ATX-101 (6 μM), AEE788 (1 μM), BafA1 (Bafilomycin A1) (10 nM) or the combination of these for 24h relative to untreated control. The average viabilities ± SD are displayed (n=3 biological replica), ^*^p<0.05 (t-test, paired).

The proteomic analysis indicated an upregulation of endocytosis, lysosomal events and autophagy by the combination treatment (Figure 4A, 4B). This suggests that altered regulation of vesicular trafficking and degradation of cellular components are important molecular consequences of the treatment. To explore this further, we evaluated the effects on autophagy, both basal levels and levels after inhibition (bafilomycin A1 inhibits the fusion of autophagosomes and lysosomes) (Figure 5A). LC3 is converted from LC3-I to LC3-II upon initiation of autophagy, thus, an increased LC3-II/LC3-I ratio, as seen by the combination treatment (Figure 5B, upper panel), can indicate increased autophagy. Both LC3-II and p62 bind to the membrane of autophagosomes. During fusion with lysosomes in the late stage of autophagy, p62 is degraded while LC3-II is released. Thus, increased LC3-II and p62 after inhibition of this fusion with bafilomycin A1 are indicative of increased autophagic flux [30]. We found that basal levels of LC3-II were slightly increased in both AEE788 and combination treated cells relative to untreated control, while basal levels of p62 were unchanged in all treatments. However, the combination treated cells had the largest accumulation of both LC3-II and p62 after autophagy inhibition, supporting increased autophagy (Figure 5B, lower panel). Corresponding measurements showed a small reduction in viability after 24h when autophagy was inhibited in combination with the ATX-101/AEE788 treatment (Figure 5C). Cumulatively these results suggest that the autophagic flux is increased in ATX-101/AEE788 treated cells compared to either single agent treatment, in agreement with the observations made from MIB-assay (Figure 4B), and that this is a response that reduce the effect of the combination treatment.

## DISCUSSION

Kinase inhibitors are actively being investigated in clinical studies. However, resistance to single inhibitors have changed the focus to combinations of inhibitors to improve treatment response [2]. This study demonstrates for the first time that the PCNA-targeting peptide ATX-101 increases the anti-cancer effects of the EGFR/HER2/VEGFR inhibitor AEE788. One of the challenges with combination therapy is that drug-drug interactions can increase side effects and reduce tolerability in patients. Previous studies with AEE788 in combination therapy showed promise in preclinical studies, however, failed in clinical trials due to unacceptable side effects [7, 31–33]. If the ATX-101/AEE788 combination, or other ATX-101/kinase inhibitor combinations have acceptable side effects remains to be explored, but no toxicity with respect to body weight and general appearance was observed in the mice of this study. Nevertheless, the purpose of this study was to examine if targeting PCNA could enhance the effect of targeted therapeutic drugs and not only chemotherapeutic drugs, as shown previously [21, 23–25].

Emerging results suggest that PCNA is central in many stress-related pathways in addition to DNA repair, e.g. apoptosis, immune evasion and cellular signaling [9, 10, 12, 17, 34]. APIM-containing proteins seem to have a higher affinity to modified PCNA after cellular stress [18, 35]. Thus, ATX-101 utilizes nature’s own regulation of PCNA affinities, with minimal effects on essential PCNA functions such as replication [17, 18, 21, 23]. Furthermore, ATX-101 partly impairs multiple pathways simultaneously without the need for multiple different inhibitors. The heterogeneity of for example breast cancer subtypes such as triple negative breast cancer (TNBC) makes treatment challenging [36], and TNBC may in particular benefit from a broad targeted strategy. Because AEE788 induces cellular stress by blocking EGFR/HER2/VEGFR signaling and not by damaging the DNA, and because ATX-101 has previously been shown not to affect cell cycle distribution [21, 25], it is likely the cytosolic roles of PCNA that are important in this context. As a side note, we did observe a substantial amount of PCNA also in the cytosol of 67NR cells (unpublished data). We have previously shown that combining p38 MAPK inhibition with the APIM-peptide enhanced growth inhibition in both human and yeast cells and reduced cytokine production from monocytes [17, 37]. The results shown here further support a cytosolic scaffolding role of PCNA in cellular signaling.

Our proteome analysis indicated that Akt and MAPK signaling were upregulated in the ATX-101/AEE788 combination treated cells compared to AEE788 treated cells. These pathways are generally associated with tumor progression and cell survival. However, recent data suggests that one of the mechanisms inducing chemoresistance and progression in breast cancer is driven by chemokine receptor CXCR2 overexpression, causing Akt1 suppression and COX2 activation [38]. This suggests that upregulation of Akt1 and simultaneously downregulation of COX2, as observed in our data set, may actually be beneficial in breast cancer. COX2 overexpression is associated with progression in multiple cancers, and COX2 inhibitors have shown promising therapeutic effects in clinical trials [39, 40]. Furthermore, both GRB2 and MEK5/ERK5 are commonly overexpressed in breast cancer and associated with poor overall survival, and targeting of these proteins/pathways is shown to suppress breast cancer progression [41–43]. Thus, downregulation of COX2, GRB2 and MEK5 as seen in the ATX-101/AEE788 combination treatment supports the phenotype observed in this pre-clinical breast cancer model.

Our data suggest that autophagic flux is increased as a response to ATX-101/AEE788 combination treatment. Autophagy is considered a two-edged sword in cancer as it may contribute to apoptosis, but also provide the cancer cells with recycled cellular components needed to sustain its growth. The role of autophagy in breast cancer is likely dependent on subtype, context and cancer stage. Downregulation of the important autophagic proteins ULK and Beclin-1 often occurs in breast cancer, particularly in TNBC, suggesting that activation of autophagy may be beneficial [44]. Other studies suggest that a molecular switch is deciding the autophagy-mediated fate of the cells, and that increased autophagy above a threshold will force autophagic cell death [44-46]. As AEE788 is previously published to induce the pro-survival activity of autophagy [47], the observed increase in autophagic flux in our study could be an attempt to overcome the therapeutic stress. The reduced viability detected at 24h when ATX-101/AEE788 was combined with an autophagy inhibitor, suggest an initial increased autophagic flux which reduce the effect of the ATX-101/AEE788 treatment. However, an enhanced anti-cancer effect when combining ATX-101 with AEE788 treatment was still detected. The multiple theoretical effects of targeting PCNA with ATX-101 makes it difficult to predict the most prominent downstream effects, and it is likely the combination of multiple effects that results in the increased anti-cancer phenotype observed.

## MATERIALS AND METHODS

### Cell lines

The 67NR mouse mammary tumor cell line were kindly provided by Fred Miller (Wayne State University, Detroit, MI). 67NR cells are derived from a spontaneous tumor in a Balb/cfC3H mouse and are HER2-/PR- and ER+/EGFR+ [27, 28]. Cells were cultured as described [48]. The human bladder cancer cell line 5637 (ATCC No. TCP-1020) and the human colon cancer cell line SW480 were cultured in RPMI 1640 media, while the human breast cancer cell line MDA-468 were kindly provided by professor Berit Johansen (Norwegian University of Science and Technology, Norway), and cultured in Dulbecco’s Modified Eagle Medium (Sigma-Aldrich). 5637, SW480 and MDA-468 are all EGFR-overexpressing cell lines.

### Viability assay

Cell growth over time was measured using the PrestoBlue Cell Viability Reagent (Invitrogen). Briefly, 67NR, 5637, SW480 or MDA-468 cells were seeded in 96-well plates (2,500 cells/well) and exposed to ATX-101 (25 amino acid cell-penetrating APIM-containing peptide [21], Ac-MD-RWLVK-W-KKKRK-I-RRRRRRRRRRR, Invitrogen, Sweden) (4-12 μM), ATX-A (Ac-MD-RALVK-W-KKKRK-I-RRRRRRRRRRR, Invitrogen, Sweden) (6 μM), AEE788 (EGFR/HER2/VEGFR inhibitor, Selleck Chem) (0.5-1 μM), Bafilomycin A1 (10 nM) Cell Signaling, #54645, or cMet inhibitor (PHA-665752, Sigma, 2 μM) until harvest at day three. Data displayed are percentage viability relative to untreated control in one representative experiment out of three biological replicas demonstrating the same trend.

### Animals and ethics

Animal experiments were performed at the Unit of Comparative Medicine, NTNU and approved by the Norwegian Food Safety Authority (FOTS applications 7448) in accordance with Norwegian and EU guidelines for care and use of laboratory animals. Female BALB/cfC3H mice (8 weeks, Taconic, Rensselaer, NY, USA) were kept in a standardized environment and monitored for health status throughout the experiments.

### Orthotopic mammary cancer model in mice

67NR cells were injected into the mammary fat pads of mice at day 0 as described [48]. The mice were treated 3x/week (up to six treatments) from day 7 with vehicle (97% polyethylene glycol 300 (PEG300, Merck) in DMSO (V/V), per oral (p.o), 0.15 mL, n=11), ATX-101 (6 mg/kg net peptide, intraperitoneal (i.p), 0.2 mL, n=11)(APIM Therapeutics, Trondheim, Norway), AEE788 (25 mg/kg, p.o, 0.15 mL, n=16) or APIM-peptide/AEE788 combination (n=16). Tumors were measured (3x/week, electronic Vernier Caliper) and volumes calculated using the formula for a spheroid: $\frac{4}{3}\times a^{2}\times b\times \pi$ (2a=tumor width, 2b=tumor height). A subgroup of these mice were harvested 24 hours after the third treatment (n=6 in each treatment group). The tumors from these mice were used to study the kinome using the multiplexed-inhibitory bead (MIB)-assay. The remaining mice were used to follow tumor growth and overall survival (vehicle: n=5, ATX-101: n=5, AEE788: n=10, combination: n=10). Mice were euthanized using carbon dioxide (2L/min) and tumors harvested when they reached their humane end point (judged by tumor burden and health status). Treatments were stopped when tumors reached 900 mm^3^ or at day 19.

### MIB-assay

Breast tumors harvested for kinome/MIB-assay studies were homogenized before protein extraction. Additionally, protein extracts were collected from 67NR cells (1.5x10^6^) grown for 24 hours before treatment with AEE788 (1 μM), ATX-101 (6 μM) or the combination of these for additional 24 hours (n=3 for each treatment group). Proteins were extracted using Mammalian Protein Extraction Reagent (Thermo Fischer Scientific) according to the manufacturer’s instructions and with Halt Protease inhibitor cocktail (EDTA free) and Halt Phosphatase inhibitor cocktail (Thermo Fischer Scientific) added. The final protein concentrations were adjusted to 1 mg/mL.

The MIB-assay enriching for kinases was performed using three different kinase inhibitors (Purvalanol B (Tocris Bioscience), Bisindolylmaleimide X (Activate Scientific) and SB6-060-05 [49]) immobilized on ECH sepharose 4B and EAH sepharose 4B beads (GE healthcare) as described [29], using 200 μL protein extract (1 mg/mL) per column with beads. Proteins in the eluates were identified using mass spectrometry (MS)-analysis (Orbitrap) as described [29].

### Mass spectrometry data analysis

The MS proteomics data have been deposited to the ProteomeXchange Consortium via the PRIDE partner repository with the dataset identifier PXD011044 [50]. Proteins were quantified by processing MS data using Max Quant v 1.5.7.0 [51]. Preview 2.3.5 (Protein Metrics Inc.) was used to inspect the raw data to determine optimal search criteria (Acetylation of Protein N-terminal, Oxidation of Methionine and Deamidation of Asparagine/Glutamine) [52]. These were imported in MaxQuant (one minute window match-between-run function, 20 min overall sliding window), and further queried against the mouse proteome including isoforms (Uniprot and MaxQuant’s internal contaminants database using Andromeda built into MaxQuant). Protein/peptide identifications FDR was set to 1%. Peak abundances were extracted by integrating the area under the peak curve. Each protein group abundance was normalized by the total abundance of all identified peptides for each run, and by calculated median (unique+razor peptide ion abundances) for each protein using label free quantification (LFQ) algorithm with minimum peptides ≥ 1 [53]. LFQ values for all 67NR cell samples were log2-transformed and subjected to principal component analysis (PCA) [54] These LFQ values were log transformed with base 2 and the transformed control values were subtracted. The resulting values reflecting the change relative to control for each condition were subjected to two sided non-parametric Wilcoxon signed-rank test as implemented in MATLAB R2015a (Mathworks Inc.) in order to check the consistency in directionality of the change [55]. This non-parametric test avoids the assumption of a null distribution and is robust to outliers and extreme variations noticed in observed values. Differentially expressed (DE) proteins groups were identified at p<0.25 [55]. The same procedure was done on tumor tissue samples, but no significantly changed proteins could be identified. The Uniprot accession IDs of the significant proteins were mapped to KEGG pathways using the online KEGG mapper tool (https://www.genome.jp/kegg/mapper.html). Additionally, those identified in the combination treated group were submitted to database for annotation, visualization and integrated discovery (DAVID, 6.7) for functional annotation analysis [56].

### Western blotting

67NR cells were seeded out (1.5x10^6^ cells/15 cm dish) 24 hours before treatment with ATX-101 (6 μM) and AEE788 (1 μM) for 24 hours and Bafilomycin A1 (10 nM,) for 12 hours. Cells were lysed in Urea buffer (8 M Urea, 0.5% Triton-X, 0.1 M DTT, 1x Complete EDTA-free protease inhibitor cocktail (Roche), 2x phosphatase inhibitor cocktail 2 (Sigma-Aldrich, P5726), 2x phosphatase inhibitor cocktail 3 (Sigma-Aldrich, P0044)) (2x cell volume, 4°C, 20 min). The supernatants (total cell extracts, 50 μg protein) were blotted (polyvinylidene fluoride membranes, Immobilon, Millipore), and developed using primary antibodies against LC3 (cell signaling, #3868, 1:1000), p62 (cell signaling, #5114, 1:1000) and beta-tubulin (abcam, ab6046, 1:2000), and the fluorescently labeled goat-α-mouse 800CW and goat-α-rabbit 680RD secondary antibodies (LI-COR, 1:25000). Proteins were visualized in Odyssey infrared imaging system (LI-COR Biosciences) and quantified in Odyssey Image Studio (V2.0). Data displayed are normalized against β-tubulin, relative to untreated control and average ± SD (n=3 biological replica).

## CONCLUSIONS

In this study, we show for the first time that targeting PCNA can increase the anti-cancer efficacy of targeted therapies. Our data are supportive of PCNA having important non-canonical cytosolic roles. Modifications to the kinome and increased autophagy are important cellular events following the ATX-101/AEE788 combination treatment, ultimately reducing cancer cell survival and tumor load.

## Abbreviations

HER2/ERBB2: human epidermal growth factor receptor 2
TNBC: triple negative breast cancer
VEGFR: vascular endothelial growth factor receptor
EGFR: epidermal growth factor receptor
MAPK: mitogen-activated protein kinase
PI3K: the phosphoinositide 3-kinase
PCNA: proliferating cell nuclear antigen
PIP-box: PCNA-interacting peptide box
APIM: AlkB homologue 2 PCNA-interacting motif
TLS: translesion synthesis
MIB: multiplexed inhibitor bead
PCA: Principal component analysis
ERAD: ER-associated degradation
